# Molecular Epidemiology and Genetic Profile of Non‐Typhoidal *Salmonella* Serovars Isolated From Poultry Production Chain in Khorasan Province, Iran: A Comprehensive Analysis of Prevalence, Virulence Genes, and Antibiotic Resistance

**DOI:** 10.1002/vms3.70757

**Published:** 2026-01-06

**Authors:** Ali. Zavari, Mahdi. Askari Badouei, Gholamreza. Hashemi Tabar

**Affiliations:** ^1^ Department of Pathobiology Faculty of Veterinary Medicine Ferdowsi University of Mashhad Mashhad Iran

**Keywords:** antimicrobial resistance, non‐typhoidal *Salmonella*, poultry production chain, public health concern, virulence factors

## Abstract

Non‐typhoidal *Salmonella* (NTS) serovars are important foodborne zoonotic pathogens that threaten human health and contribute to the evolution and global dissemination of antibiotic resistance. This study investigated the virulence characteristics, antibiotic susceptibility, serovar distribution, and antimicrobial resistance determinants of 132 NTS isolates obtained from the poultry production chain in Khorasan Province, Iran, between 2018 and 2020. *Salmonella* Infantis was the predominant serovar, accounting for 53.8% of isolates, followed by *S*. Enteritidis (20.5%), *S*. Typhimurium (3.8%), and 22% of isolates could not be typed by multiplex PCR and were categorized as non‐typed *Salmonella*. Based on antibiotic susceptibility tests, most *Salmonella* isolates exhibited high resistance to tiamulin (97%), flumequine (81.1%), ampicillin (96.2%), and streptomycin (78.8%). A total of 65 multidrug‐resistant (MDR) profiles were identified. Conversely, gentamicin (97%), ciprofloxacin (94%), enrofloxacin (90.9%), cefotaxime (89.4%), and colistin (88.6%) demonstrated the greatest effectiveness against these NTS isolates. The most frequently identified antimicrobial resistance genes (ARGs) were *bla_TEM_* (91.7%), *sul1* (80.3%), and *aadA* (78.8%). In addition, PCR analysis of virulence genes showed that all isolates harbored the *stn* and *iroN* virulence genes. However, *pefA* and the *Salmonella* plasmid virulence genes (*spvB*, *spvC*, and *spvR*) were detected in 53% and 36.4% of the isolates, respectively. In conclusion, our findings highlight the increasing prevalence of MDR *S*. Infantis, which has replaced *S*. Enteritidis and *S*. Typhimurium as the dominant serovars in the poultry production chain. Effective control of NTS in poultry production chain requires regular monitoring and surveillance of NTS infections, serotype diversity, and antimicrobial resistance profiles.

## Graphical abstract

This study presents a comprehensive overview of the prevalence of main serovars, phenotypic and genotypic antimicrobial resistance, and virulence factors of non‐typhoidal *Salmonella* (NTS) serovars isolated from various stages of the poultry production chain, as well as their importance to human health in Iran.

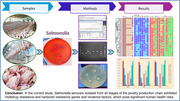

## 1 Introduction

*Salmonella* genus contains rod‐shaped, Gram‐negative bacteria belonging to the family *Enterobacteriaceae*. *Salmonella* species are categorized into typhoidal and non‐typhoidal based on their capacity to induce specific human diseases (Billah and Rahman 2024). Non‐typhoidal *Salmonella* (NTS) is a significant human foodborne pathogen, responsible for approximately 153 million cases of gastroenteritis and 57,000 deaths annually (Han et al. 2024). The broad host spectrum of NTS explains the global prevalence of NTS serovars such as *Salmonella* Typhimurium and *S*. Enteritidis. Human non‐typhoidal salmonellosis is often a self‐limiting disease. However, it may become severe in vulnerable individuals, such as children, the elderly, and immunocompromised people (Kumar et al. 2025; Lamichhane et al. 2024). NTS serovars are found in the gastrointestinal tracts of different warm‐blooded and cold‐blooded animals, including livestock, birds, pets, and wildlife (Han et al. 2024; McDermott et al. 2018). Recent studies have proposed that certain NTS serovars associated with invasive NTS disease in sub‐Saharan African countries use humans as their reservoir (Hagedoorn et al. 2023).

Humans acquire NTS infections primarily through the consumption of contaminated food, fruits, vegetables, and water, as well as through contact with infected host animals. Poultry meat and eggs are the principal sources of human salmonellosis. Furthermore, ingestion of contaminated beef, pork, lamb, goat meat, dairy products, and vegetables also causes human infections (Lu et al. 2025; Yörük 2025; Zavari et al. 2025).

Today, managing NTS infections has become more challenging due to the emergence of antibiotic‐resistant strains. Antibiotic resistance in NTS is increasing globally, with some strains developing resistance to multiple classes of antibiotics (Kumar et al. 2025; McDermott et al. 2018). Antimicrobial resistance (AMR) is among the top ten public health concerns, posing a serious threat to human health (EClinicalMedicine 2021). According to predictive statistical models, AMR in pathogenic bacteria was responsible for approximately 1.27 million deaths worldwide in 2019 (Ho et al. 2025). The misuse and overuse of antibiotics in human and animal health have contributed to the development of AMR (Angeles‐Hernandez et al. 2025; Qin et al. 2022). Surveillance of AMR plays a critical role in guiding clinical decisions. Additionally, it enables public health authorities to monitor drug resistance patterns, evaluate the effectiveness of interventions, and implement control measures (Ho et al. 2025). Consequently, several AMR monitoring programs, such as the World Health Organization (WHO) Global Plan of Action (GAP), the European Union's One Health Action Plan against AMR, and the Central Asian and Eastern European Regions (CAESAR network), have been established (Pandey et al. 2022).

According to the Kauffmann–White‐Le Minor classification, more than 2500 *Salmonella* serovars have been recognized based on somatic (O) and flagellar (H) surface antigens (Hagedoorn et al. 2023). The distribution and severity of *Salmonella* serovars differ according to geographic location and may change over time. For example, the most prevalent serovars of human infection are *S*. typhimurium in Australia, *S*. Enteritidis in Brazil, and *S*. Enteritidis, *Salmonella* Newport, *S*. Typhimurium, and *Salmonella* Javaiana in the United States. Similarly, in the European Union, *S*. Enteritidis, *S*. Typhimurium, *S*. Infantis, and *Salmonella* Newport are the most common *Salmonella* serovars (Cheng et al. 2019; EFSA 2023; Katiyo et al. 2019; McDermott et al. 2018). In Iran, *S*. Enteritidis and *S*. Typhimurium have been the most prevalent. Recent studies have also identified *S*. Infantis (Badouei et al. 2021; Khademi et al. 2020; Ranjbar et al. 2018; Rezaei et al. 2022).

Iran is one of the major poultry producers in the Middle East, and poultry meat and eggs are a primary source of protein for its population, as well as an export commodity to other countries. This study was conducted in Khorasan province, one of the primary regions for chicken and egg production in Iran. In this study, we investigated NTS serovars isolated from different stages of the poultry production chain and identified the predominant serovars. We also assessed their phenotypic resistance to antibiotics commonly used in human and veterinary medicine. To evaluate the potential pathogenicity of NTS isolates, we analysed virulence factors associated with bacterial colonization, enterotoxicity, intracellular survival, replication, and systemic dissemination. Furthermore, plasmid‐borne genes conferring resistance to antibiotics and contributing to the evolution and dissemination of AMR were examined.

## 2 Materials and Methods

### 2.1 Bacterial Strains

A total of 132 *Salmonella* isolates were examined in this study. These isolates had been previously recovered from different stages of the poultry production chain through government and non‐government surveillance programs. Isolation had been performed between 2018 and 2020 in laboratories located in Razavi Khorasan and South Khorasan provinces in Northeastern and Eastern Iran, following the ISO 6579‐1 standard protocol. All isolates were preserved in Brain Heart Infusion (BHI) Broth (HiMedia, India) containing 20% glycerol at −80°C. After recovery, the isolates were phenotypically confirmed to belong to the *Salmonella* genus based on colony morphology and biochemical characteristics on Xylose Lysine Desoxycholate (XLD) Agar (Condalab, Spain), Triple Sugar Iron (TSI) Agar (Condalab, Spain), Urea Agar (Condalab, Spain) and Lysine Decarboxylase Broth (Condalab, Spain). A single colony from each isolate was then selected and cultured on Nutrient Agar (Condalab, Spain). Antibiotic susceptibility testing, molecular confirmation, and analyses of virulence and resistance genes were performed on the subcultured colonies. Details of the NTS isolates investigated in this study are provided in Supporting Information S1.

### 2.2 Antimicrobial Susceptibility Testing

All 132 *Salmonella* isolates were evaluated for AMR by the disk diffusion assay according to the 2021 Clinical and Laboratory Standards Institute (CLSI) guidelines (CLSI 2021; Weinstein 2018). The criteria suggested by García‐Meniño et al. (2020) were applied for colistin (CT). The concentrations of the antibiotic discs were as follows: ampicillin (AMP; 10 µg), gentamicin (GN; 10 µg), CT (10 µg), tetracycline (TE; 30 µg), streptomycin (S; 10 µg), trimethoprim/sulfamethoxazole (SXT; 25 µg), ciprofloxacin (CIP; 5 µg), neomycin (N; 30 µg), cefotaxime (CTX; 30 µg), tiamulin (TM; 30 µg), enrofloxacin (NFX; 5 µg), flumequine (FM; 30 µg) and, florfenicol (FF; 30 µg) (Padtantab, Iran). Notably, the final four antibiotic discs were exclusively used for veterinary purposes. Multidrug resistance (MDR) was defined as resistance to three or more classes of antibiotics (McDermott et al. 2018). *Escherichia coli* ATCC 25922 was employed as the bacterial control strain for antibiotic susceptibility testing.

### 2.3 DNA Extraction and Molecular Confirmation

The High Pure PCR Template Preparation Kit (Roche, Germany) was used to extract and purify DNA from fresh cultures of *Salmonella* isolates, following the kit's instruction manual. The purity and quantity of the extracted DNA were examined using agarose gel electrophoresis and spectrophotometry on a Biotek Epoch microplate spectrophotometer with Gen5 software. According to Hoorfar et al. (2000), Real‐time PCR techniques were employed to confirm that all NTS isolates belonged to the *S. enterica* subspecies. Each real‐time PCR reaction contained 12.5 µL of Real Q Plus 2× Master Mix for Probe (Ampliqon, Denmark), 400 nmol of each primer, 250 nmol of a TaqMan probe (Metabion, Germany), and 3 µL of template DNA. Real‐time PCR and multiplex PCR products were sent for sequencing (Macrogen, South Korea), and the results were aligned and verified using the NCBI BLAST tool. Supporting Information S2 lists the sequences of all primers and the TaqMan probe utilized in this study, as well as details of the PCR tests, including the target gene, annealing temperature, and amplicon size.

### 2.4 Molecular Serovar Typing

*S*. Enteritidis and *S*. Typhimurium are two prevalent NTS serovars in humans and poultry in Iran and other regions. Recent studies have identified *S*. Infantis as an emerging pathogen widespread globally in the poultry industry (Badouei et al. 2021; Cheng et al. 2019; McDermott et al. 2018; Ranjbar et al. 2018; Rezaei et al. 2022). We examined the prevalence of these three important NTS serovars through multiplex PCR using three sets of specific primer pairs. Multiplex PCR was conducted in a Veriti Thermal Cycler machine (Applied Biosystems, USA) with a total reaction volume of 25 µL, consisting of 12.5 µL of Multiplex TEMPase 2× Master Mix (Ampliqon, Denmark), 0.8 µL of each primer (10 µM solution), 4.7 µL of PCR‐grade distilled water (Sinaclone, Iran), and 3 µL of extracted template DNA. Amplified products were analysed by electrophoresis on a 1.5% agarose gel stained with DNA Safe Stain (Sinaclone, Iran).

### 2.5 Determination of Antimicrobial Resistance Genes

*Salmonella* isolates were evaluated by simplex PCR for the presence of eight Antimicrobial Resistance Genes (ARGs). Three β‐lactam resistance genes (*bla_TEM_*, *bla_SHV_*, and *bla_OXA_*), two tetracycline resistance genes (*tetA* and *tetK*), an aminoglycoside resistance gene (*aadA*), a sulfonamide resistance gene (*sul1*), and a trimethoprim resistance gene (*dhfrV*) were evaluated. All simplex PCRs were performed in 20 µL volumes using 10 µL of Taq DNA Polymerase 2× Master Mix RED (Ampliqon, Denmark), 1 µL of each forward and reverse primer (10 µM solution), 5 µL of PCR‐grade water, and 3 µL of extracted template DNA. PCR thermal cycling was run in Applied Biosystems Veriti Thermal Cycler under the following programs: initial denaturation at 95°C for 3 min; 35 cycles of denaturation (95°C for 30 s), annealing (at the ARGs specific temperature for 40 s, see Supporting Information S2), and extension (72°C for 1 min); finally, a single cycle of extension at 72°C for 5 min.

### 2.6 Determination of Virulence Determinants

*Salmonella* isolates were evaluated for the presence of six virulence determinants. Three *Salmonella* plasmid virulence genes (*spvB*, *spvC*, and *spvR*), the plasmid‐encoded major fimbrial subunit (*pefA*), and two chromosomal virulence factors, *Salmonella* enterotoxin (*stn*) and the siderophore receptor (*iroN*), were investigated. The prevalence of virulence determinants in all isolates was determined using the Simplex PCR technique with utilized Taq DNA Polymerase Master Mix RED (Ampliqon, Denmark) and specific primer pairs (10 µM solution), similar to the protocol employed for detecting resistance genes.

### 2.7 Statistics and Clustering

We utilized IBM SPSS Statistics (version 26) to conduct a chi‐square test with a 95% confidence interval to evaluate the relationship between the presence of ARGs and phenotypic resistance to the corresponding antibiotics in NTS isolates. A *p* value of less than 0.05 indicated a statistically significant association between the resistance phenotype and the presence of the corresponding ARGs. A hierarchical clustering heatmap was created using the ComplexHeatmap package in R software (version 4.3.0).

## 3 Results

### 3.1 Antimicrobial Susceptibility Patterns

**FIGURE 1:**
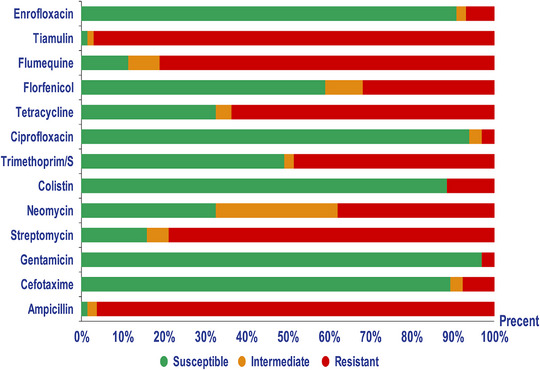
Antimicrobial susceptibility testing of non‐typhoidal *Salmonella* (NTS) isolates showed significant resistance to ampicillin, tiamulin, streptomycin, and flumequine (red). Conversely, fluoroquinolones, gentamicin, cefotaxime, and colistin demonstrated the highest effectiveness against NTS isolates (green).

The present study evaluated the susceptibility of 132 *Salmonella* isolates to different classes of antibiotics. Most NTS isolates were susceptible to GN (97%), ciprofloxacin (94%), enrofloxacin (90.9%), cefotaxime (89.4%), and CT (88.6%). In contrast, a majority of NTS isolates exhibited MDR patterns. Notable resistance was observed to tiamulin (97%), AMP (96.2%), and streptomycin (78.8%). *S*. Enteritidis was more sensitive to TE (11.1%), trimethoprim‐sulfamethoxazole (14.8%), and streptomycin (44.4%) than other serovars. Whereas *S*. Infantis isolates were more resistant to these antibiotics. Details are provided in Figure 1 and Table 1. Among the NTS isolates, 67 specific antibiotic resistance patterns were identified, 65 of which were classified as MDR. The most common MDR profile, observed in nine isolates, included resistance to AMP, streptomycin, flumequine, and trimethoprim (Supporting Information S1).

**TABLE 1:** The phenotypic antimicrobial resistance in non‐typhoidal *Salmonella* isolates of poultry origin from 2018 to 2020, categorized by serovar.

	No. (%). of resistance isolates of each serovars				
Antibiotics	*S*. Enteritidis (*N* = 27)	*S*. Infantis (*N* = 71)	*S*. Typhimurium (*N* = 5)	Non‐typed (*N* = 29)	Total (*N* = 132)
gentamicin (GN)	0	0	0	0	0
ampicillin (AMP)	26 (96.3)	69 (97.2)	5 (100)	27 (93.1)	127 (96.2)
streptomycin (S)	12 (44.4)	63 (88.7)	5 (100)	24 (82.8)	104 (78.8)
neomycin (N)	14 (51.9)	26 (36.6)	1 (20)	9 (31)	50 (37.9)
cefotaxime (CTX)	0	8 (11.3)	0	2 (6.9)	10 (7.6)
colistin (CT)	1 (3.7)	7 (9.86)	2 (40)	5 (17.2)	15 (11.4)
trimethoprim/sulfamethoxazole (SXT)	4 (14.8)	49 (69)	1 (20)	10 (34.5)	64 (48.5)
ciprofloxacin (CIP)	1 (3.7)	3 (4.2)	0	0	4 (3)
tetracycline (TE)	3 (11.1)	59 (83.1)	4 (80)	18 (62.1)	84 (63.6)
florfenicol (FF)	4 (14.8)	29 (40.8)	0	9 (31)	42 (31.8)
tiamulin (TM)	26 (96.3)	70 (98.6)	5 (100)	27 (93.1)	128 (97)
flumequine (FM)	23 (85.2)	57 (80.3)	2 (40)	25 (86.2)	107 (81.1)
enrofloxacin (NFX)	0	7 (9.9)	0	2 (6.9)	9 (6.8)
co‐resistance to CTX/CIP	0	1 (1.4)	0	0	1 (0.8)

### 3.2 Molecular Confirmation and Serovar Typing

All 132 *Salmonella* isolates were identified as *S. enterica* through a real‐time PCR test that targeted the *invA* gene. A multiplex PCR assay was then established to detect the three most prevalent *Salmonella* serovars in the poultry production chain in Iran. Multiplex PCR results showed that *S*. Infantis was the most common serovar (53.8%), followed by *S*. Enteritidis (20.5%) and *S*. Typhimurium (3.8%). Based on the negative multiplex PCR results, 29 isolates (22%) were classified as non‐typed *Salmonella* serovars. In our study, as summarized in Table 2, *S*. Infantis was the predominant serovar isolated from chicken carcasses and chicken paste, while *S*. Enteritidis was the major serovar isolated from poultry farms. Additionally, *S*. Typhimurium was only identified in chicken carcasses and slaughterhouse isolates.

**TABLE 2:** Percentage prevalence of non‐typhoidal *Salmonella* serovars across the poultry production chain.

Source (no. isolated)	*S*. Enteritidis (*N* = 27)	*S*. Typhimurium (*N* = 5)	*S*. Infantis (*N* = 71)	Non typed (*N* = 29)
Poultry farm (*N* = 13)	8 (61.5)	0 (0)	3 (23)	2 (15.4)
Slaughterhouse (*N* = 6)	0 (0)	1 (16.7)	4 (66.6)	1 (16.7)
Poultry carcass (*N* = 83)	16 (19.3)	3 (3.6)	49 (59)	15 (18)
Chicken paste (*N* = 9)	1 (11)	0 (0)	6 (66.7)	2 (22.2)
Grind meat (*N* = 21)	2 (9.5)	1(4.8)	9 (42.8)	9 (42.8)

### 3.3 ARGs Profiles

**FIGURE 2:**
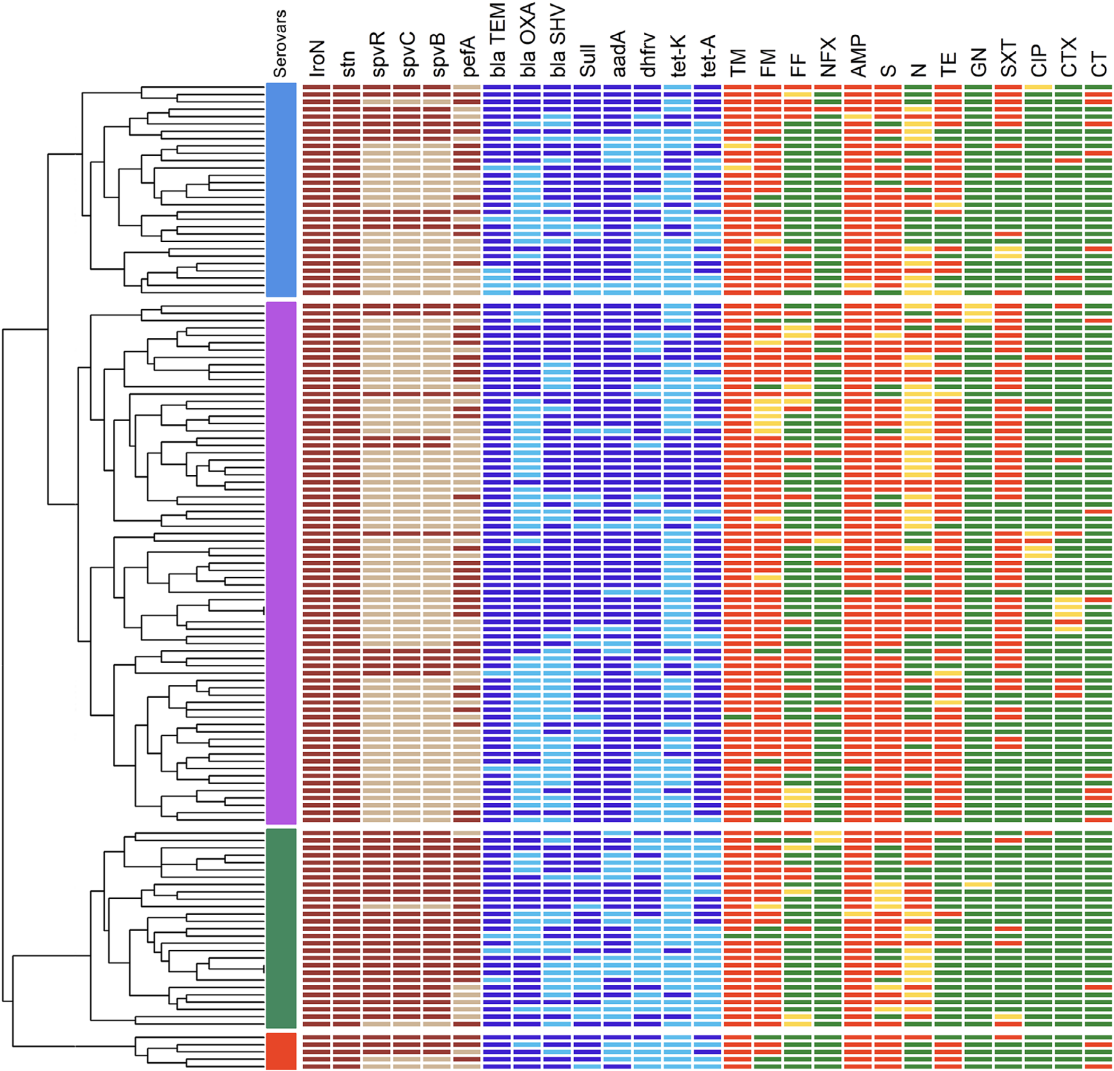
The hierarchical clustering heatmap of non‐typhoidal *Salmonella* isolates includes virulence factors, antimicrobial resistance genes (ARGs), and phenotypic antimicrobial resistance (AMR). The first column after the dendrogram displays the serovars: *Salmonella* Typhimurium (red), *Salmonella* Enteritidis (green), *Salmonella* Infantis (purple), and non‐typed serovars (blue). Dark brown indicates the presence of virulence factors, while light brown represents their absence. Notably, *Salmonella* Enteritidis possesses a higher number of virulence factors. Additionally Dark blue indicates the presence of ARGs, while light blue represents the absence of ARGs (blaTEM is the most prevalent ARG in all NTS serovars). Light green indicates susceptibility, orange represents intermediate resistance, and red signifies resistant phenotypes of NTS isolates to antibiotics. Ciprofloxacin, gentamicin, cefotaxime, and enrofloxacin show higher susceptibility rates. (AMP, ampicillin; CIP, ciprofloxacin; CT, colistin; TE, tetracycline; CTX, cefotaxime; FF, florfenicol (NFX, 5 µg), flumequine; GN, gentamicin; N, neomycin; NFX, enrofloxacin; S, streptomycin; SXT, trimethoprim/sulfamethoxazole; TM, tiamulin).

All 132 NTS isolates were analysed for the presence of eight ARGs associated with phenotypic AMR. *Bla_TEM_* (91.7%), *sul1* (80.3%), and *aadA* (78.8%) were the most common, while the TE resistance gene (*tetK*) had the lowest incidence rate at 28%. All *S*. Typhimurium strains (100%) harboured the *bla_TEM_* gene. Furthermore, *S*. Typhimurium exhibited a higher prevalence of three β‐lactam resistance genes (*bla_TEM_*, *bla_OXA_*, and *bla_SHV_*) than *S*. Infantis, *S*. Enteritidis, and the non‐typed serovar (Table 3, Figure 2). Three *S*. Infantis isolates, and one non‐typed isolate contained all eight resistance genes. Statistical analyses demonstrated a significant correlation between the presence of *bla_TEM_*, *tetA*, and *aadA* genes and phenotypic resistance to AMP (*p *= 0.027), TE (*p *= 0.000), and streptomycin (*p *= 0.001). However, no significant associations were found between other resistance genes and their corresponding antibiotics (*p* > 0.05).

**TABLE 3:** Prevalence of antibiotic resistance genes in different non‐typhoidal *Salmonella* serovar isolated from poultry production chain.

Salmonella serovar (number)	Prevalence of antibiotic resistance genes (No/%)							
	blaTEM	blaOXA	blaSHV	Sull	aadA	dhfrv	tet‐K	tet‐A
*S*. Enteritidis (*N* = 27)	24 (88.9)	17 (63)	14 (51.9)	17 (63)	14 (51.9)	11 (40.7)	4 (14.8)	6 (22)
*S*. Infantis (*N* = 71)	69 (97.2)	31 (43.7)	42 (59.2)	61 (85.9)	65 (91.5)	48 (67.6)	24 (33.8)	61 (85.9)
*S*. Typhimurium (*N* = 5)	5 (100)	4 (80)	4 (80)	4 (80)	2 (40)	1 (20)	0 (0)	2 (40)
Non‐Typed (*N* = 29)	23 (79.3)	14 (48.3)	20 (69)	24 (82.8)	23 (79.3)	10 (34.5)	9 (31)	15 (51.7)
Total (*N* = 132)	121 (91.7)	66 (50)	80 (60.6)	106 (80.3)	104 (78.8)	70 (53)	37 (28)	84 (63.6)

### 3.4 Virulence Factor Profiles

**FIGURE 3:**
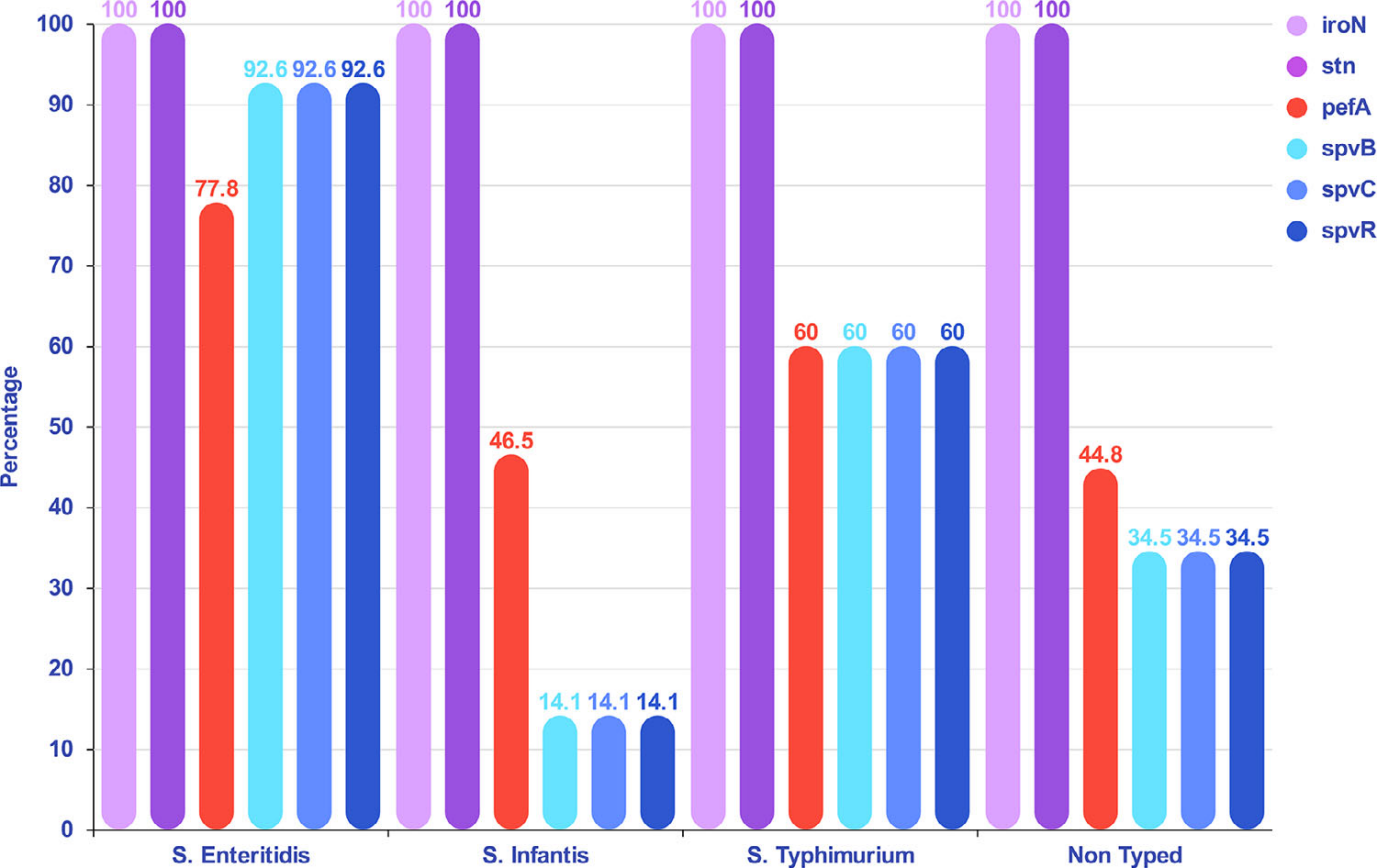
The distribution of virulence genes in *Salmonella* serovars reveals that *Salmonella* Enteritidis isolates exhibit a higher prevalence of virulence genes, especially the *spv* genes (*spvB*, *spvC*, and *spvR*), in comparison to other serovars. Conversely, the lowest distribution of *spv* genes was observed in *Salmonella* Infantis isolates.

All NTS isolates contained two virulence factors: *stn* (100%) and the *iroN* (100%). However, other virulence factors, such as the major fimbrial subunit plasmid (*pefA*, 53%) and the *Salmonella* virulence plasmid genes (*spvB*, *spvC*, and *spvR*), showed greater diversity among the *Salmonella* serovars. Notably, *spvB*, *spvC*, and *spvR* genes had the same prevalence of 36.4%. Nineteen *S*. Enteritidis (70.4%), two *S*. Typhimurium (40%), seven *S*. Infantis (9.8%), and five non‐typed isolates (17.2%) harboured all six virulence genes. In this study, virulence factors were most prevalent in *S*. Enteritidis, followed by *S*. Typhimurium (Figure 3).

## 4 Discussion

NTS is a major public health concern, particularly due to its association with animal‐origin foods and its significant risk to human health. Beyond causing human infections, it also results in considerable economic losses (Sanni et al. 2023). This study examined NTS infections in the poultry production chain and their implications for human health. Multiplex PCR analysis showed that *S*. Infantis was the predominant serovar (53.8%), while *S*. Typhimurium (3.8%) was the least prevalent. Notably, 29% of NTS isolates in this study did not belong to any of the three studied *Salmonella* serovars, indicating the presence of other serovars. The significant prevalence of *S*. Infantis is consistent with previous research by Badouei et al. (2021), who reported *S*. Infantis (76.6%) as the dominant serovar in broiler farms. Also, Ghodousi et al. and Manzari et al. reported prevalences of 52.0% and 51.4% for *S*. Infantis among chicken‐derived NTS isolates, respectively. Furthermore, consistent with our results, *S*. Enteritidis was identified as the second most prevalent serovar (Ghoddusi et al. 2019; Manzari et al. 2022). In contrast, Piryaei et al. (2025) found that *S*. Enteritidis was the predominant NTS serovar (85%) in broiler farms, based on PCR analysis. These differences in NTS prevalence may be due to geographic location, sample sources, isolation time, and biosafety practices. The incidence of *S*. Infantis in chicken farms and related food products in the United States and the European Union has been increasing. A high prevalence of this serovar has also been reported in Japan (72.2%), Ecuador (84%), and Turkey (91%), indicating increasing contamination of the poultry industry with *S*. Infantis in different regions (Acar et al. 2019; Mattock et al. 2024). Recently, the conjugated megaplasmid pESI has been identified in *S*. Infantis, which increases antibiotic resistance and overall fitness in this serovar, thereby contributing to the increasing prevalence of this serovar (Alvarez et al. 2023; Mattock et al. 2024). Based on previous research in Iran, *S*. Enteritidis (41%) was reported as the most prevalent serovar isolated from human disease (Manzari et al. 2022; Rezaei et al. 2022). Nevertheless, the current study has found that only 20% of NTS isolated from poultry sources belong to *S*. Enteritidis. Interestingly, *S*. Infantis has now become the predominant serovar associated with chickens, which may increase the risk of human infections with this serovar in the future. The increased prevalence of *S*. Infantis in this area may be due to several factors: (a) *S*. Infantis serovars usually harbor pESI megaplasmids that enhance the MDR and adaptability of this serovar; and (b) control programs such as vaccination that reduced other serovars like *S*. Enteritidis and *S*. Typhimurium, indirectly allowing *S*. Infantis to become dominant in poultry.

Antibiotic susceptibility testing revealed that 98.5% of *Salmonella* isolates from all stages of the poultry production chain were resistant to three or more classes of veterinary and medical antibiotics, indicating MDR. None of the NTS isolates were fully susceptible to all tested antibiotics. Notably, the highest resistance rates were observed for AMP (96.2%), streptomycin (78.8%), TE (63.6%), and trimethoprim‐sulfamethoxazole (48.5%), as well as for the veterinary drugs flumequine (81.1%) and tiamulin (97%) (Figure 1). *S*. Infantis isolates exhibited higher levels of antibiotic resistance than other *Salmonella* serovars, which is consistent with previous studies conducted in Iran (Badouei et al. 2021; Ranjbar et al. 2018). Furthermore, the highest resistance level was observed in three *S*. Infantis isolates that exhibited resistance to nine different antibiotics. These findings underscore the critical role of *S*. Infantis in the emergence and dissemination of AMR within the poultry production chain (Figure 2 and Supporting Information S1). The current study also identified greater phenotypic resistance to AMP and streptomycin compared to the findings of Khademi et al. (2020) and Rezaei et al. (2022), who assessed antibiotic resistance in human NTS isolates through meta‐analyses (previous studies in Iran) and disk diffusion assays, respectively. Ciprofloxacin and cefotaxime (third‐generation cephalosporins) are two important antibiotics for human salmonellosis, and combined resistance (co‐resistance) to both is significant (EFSA 2023). Our findings, consistent with previous research in Iran, demonstrated that fluoroquinolones remain the most effective antibiotics against NTS isolates (Badouei et al. 2021; Khademi et al. 2020). In addition, only one *S*. Infantis isolate (0.7%) exhibited phenotypic co‐resistance to cefotaxime and ciprofloxacin. In contrast, based on the minimum inhibitory concentration (MIC) method, *Salmonella* isolates from chicken carcasses in the European Union were 100% susceptible to cefotaxime but showed notable resistance to ciprofloxacin (74.4%) (EFSA 2023). Additionally, a study using agar dilution assays on 617 NTS isolates from food‐producing animals in China reported higher resistance rates to cefotaxime (52%) and ciprofloxacin (49.1%). Regional variations in antibiotic resistance patterns among NTS isolates are mainly influenced by differences in antibiotic usage and by the distribution of NTS serovars across various regions (Yang et al. 2023). In this study, despite the high prevalence of MDR (95.4%), most *Salmonella* isolates remained susceptible to ciprofloxacin, cefotaxime, and enrofloxacin. Moreover, as shown in Table 4, NTS isolates from poultry farms were more susceptible to streptomycin, cefotaxime, trimethoprim/sulfamethoxazole, TE, and florfenicol. Indeed, *Salmonella* isolates from the early stages of the poultry production chain exhibited lower antibiotic resistance than those from the later stages of the poultry production chain.

**TABLE 4:** The phenotypic antimicrobial resistance in non‐typhoidal *Salmonella* isolates, categorized by different stages of poultry production chain.

	No. (%) of resistant isolates				
Antibiotics	Chicken farm (*N* = 13)	Slaughterhouse (*N* = 6)	Chicken carcasses (*N* = 83)	Ground meat (*N* = 21)	Chicken Paste (*N* = 9)
Gentamicin (GN)	0	0	0	0	0
Ampicillin (AMP)	12 (92.3)	5 (83.3)	80 (96.4)	21 (100)	9 (100)
Streptomycin (S)	6 (46.2)	6 (100)	65 (78.3)	19 (90.5)	8 (88.9)
Neomycin (N)	6 (46.2)	4 (66.7)	30 (36.1)	9 (42.9)	1 (11.1)
Cefotaxime (CTX)	0.0	2 (33.3)	5 (6)	2 (9.5)	1 (11.1)
Colistin (CT)	1 (7.7)	0 (0)	7 (8.4)	4 (19)	3 (33.3)
Trimethoprim/sulfamethoxazole (SXT)	5 (38.5)	3 (50)	39 (47)	9 (42.9)	8 (88.9)
Ciprofloxacin (CIP)	0.0	1 (16.7)	3 (3.6)	0.0	0.0
Tetracycline (TE)	2 (15.4)	5 (83.3)	56 (67.5)	14 (66.7)	7 (77.8)
Florfenicol (FF)	3 (23.1)	3 (50)	24 (28.9)	9 (42.9)	3 (33.3)
Tiamulin (TM)	13 (100)	6 (100)	81 (97.6)	20 (95.2)	8 (88.9)
Flumequine (FM)	11 (84.6)	6 (100)	63 (75.9)	20 (95.2)	7 (77.8)
Enrofloxacin (NFX)	0.0	2 (33.3)	7 (8.4)	0.0	0.0

*S. enterica* can develop MDR, which increases hospitalization and threatens public health by limiting effective treatment options. *Salmonella* serovars adapt and survive in adverse conditions, such as those containing antibiotics, through chromosomal mutations or by acquiring necessary genes from other bacteria through horizontal gene transfer (HGT), leading to the emergence of resistant bacteria (Brown et al. 2021). HGT contributes to the global dissemination of ARGs (Zhu et al. 2025). In the present study, *bla_TEM_* (91.7%), *sul1* (80.3%), *aadA* (78.8%), and *tetA* (63.6%) were identified as the most common ARGs. Statistical analysis revealed a significant correlation between phenotypic resistance to TE, streptomycin, and AMP and the presence of the *tetA* (*p* = 0.000), *aadA* (*p* = 0.001), and *bla_TEM_* (*p* = 0.027) resistance genes. The high frequency of resistance genes in NTS isolates recovered from the poultry production chain highlights their potential role as horizontally transferable ARGs in this region. Similar findings were also observed by Dallal et al. for poultry‐derived NTS in Iran. According to their results, the *bla_TEM_* gene was more frequently found in *S*. Enteritidis isolates from chicken (71.4%) and eggs (90%) than in those from beef (25%) and lamb (50%). Their study also found lower rates of *tetA* (9%) and *sul1* (3%) compared to what was observed in the current study (Soltan Dallal et al. 2023). Another study by Nazari‐Moghadam et al. in Shahrekord, Iran, examined 36 *S*. typhimurium isolates from chickens and reported that all of them contained the *tetA* gene (100%), which represents a higher prevalence than that found in our study (63.6%). Nevertheless, the prevalence rate of the *sul1* gene was similar to our findings (Nazari Moghadam et al. 2023). In agreement with the current study, two studies conducted in the United Kingdom and China also identified *bla_TEM_*, *sul1*, *aadA*, and *tetA* as the most common resistance genes in *Salmonella* isolates (Card et al. 2016; Xu et al. 2020). Mobile genetic elements such as plasmids and phages facilitate the spread of resistance genes among *S. enterica* isolates through HGT mechanisms (Brown et al. 2021; Zhu et al. 2025). Among NTS serovars, various incompatibility plasmid groups, such as IncC, IncF, IncHI, and IncI1, have been identified. Moreover, a mosaic pESI‐like megaplasmid that harbors multiple antibiotic resistance and virulence genes has recently been recognized in *S*. Infantis (McMillan et al. 2020). Considering the high prevalence of incompatible plasmid groups in bacterial isolates from poultry in this region and the high frequency of transferable ARGs among NTS isolates observed in the current study, the dissemination of AMR via HGT represents a significant public health threat (Tohmaz et al. 2022; Vakili et al. 2025). In this study, NTS isolates from poultry slaughterhouses exhibited greater phenotypic antibiotic resistance compared to isolates from other stages of production. Moreover, drug resistance genes were most prevalent in slaughterhouse isolates. Therefore, the slaughtering stage plays an important role in the spread of antibiotic resistance (Tables 4 and 5).

**TABLE 5:** Prevalence of antibiotic resistance genes in non‐typhoidal *Salmonella* isolates from different stages of poultry production chain.

Origin of *Salmonella* isolates (number)	Prevalence of antibiotic resistance genes (No/%)							
	blaTEM	blaOXA	blaSHV	Sull	aadA	dhfrv	tet‐K	tet‐A
Chicken farm (*N* = 13)	11 (84.6)	7 (53.8)	3 (23.1)	5 (38.5)	6 (46.2)	6 (46.2)	2 (15.4)	3 (23.1)
slaughterhouse (*N* = 6)	6 (100)	5 (83.3)	2 (33.3)	6 (100)	5 (83.3)	3 (50)	3 (50)	6 (100)
Chicken carcasses (*N* = 83)	76 (91.6)	37 (44.6)	56 (67.5)	67 (80.7)	69 (83.1)	47 (56.6)	21 (25.3)	58 (69.9)
Grind meat (*N* = 21)	19 (90.5)	11 (52.4)	13 (61.9)	20 (95.2)	16 (76.2)	9 (42.9)	7 (33.3)	10 (47.6)
Chicken paste (*N* = 9)	9 (100)	6 (66.7)	6 (66.7)	8 (88.9)	8 (88.9)	5 (55.6)	4 (44.4)	7 (77.8)

Virulence factors in NTS, such as flagella, fimbriae, toxins, pathogenicity islands, and plasmids, determine the ability of different serovars to infect diverse hosts and cause disease. However, the prevalence of these pathogenicity factors varies among NTS serovars. As a result, the presence or absence of various virulence factors affects the ability of an isolate or a particular serovar to cause disease and infect different hosts. (Cheng et al. 2019). In this research, as shown in Figures 2 and 3, all NTS isolates harbored two chromosomal virulence factors: the *iroN* and the *stn*. These findings are consistent with a previous study that reported a high occurrence of *stn* and *iroN* (Guo et al. 2023; Kim and Lee 2017) and propose that these genes play essential roles in bacterial survival and enterotoxic activity during intestinal infection. In contrast, the distribution of plasmid‐encoded factors *pefA* (53%) and the *spv* locus (*spvB*, *spvC*, *spvR*; 36.4% each) exhibited significant variability among NTS isolates. The incidence of the major fimbrial subunit plasmid gene *pefA* (53%), which facilitates intestinal colonization through adhesion to intestinal epithelial cells, was consistent with the findings reported by Kim and Lee (2017) in Korea. Conversely, Card et al. (2016) reported a lower frequency of *pefA* in the United Kingdom (13.5%). The *spv* gene cluster (*spvB*, *spvC*, and *spvR*) was detected in 36.4% of isolates, suggesting that only a subset of NTS strains harbours the spv virulence plasmid genes that are essential for intracellular survival, replication, and systemic dissemination (Guiney and Fierer 2011; Rychlik et al. 2006). However, several studies have reported different prevalence rates of *spv* genes among NTS serovars. In Iran, Derakhshandeh et al. (2013) reported the prevalence of *spvB*, *spvC*, and *spvR* genes in NTS isolates to be 43.3%, 73.3%, and 46.6%, respectively. Similarly, Card et al. (2016) reported the prevalence of *spvC* and *spvR* at 21.8% and 20.8%, respectively, in *Salmonella* isolates in the United Kingdom. In the present study, the spv and *pefA* virulence determinants were most prevalent in *S*. Enteritidis and *S*. typhimurium. Among the examined serovars, *S*. Enteritidis exhibited the highest virulence gene content, with most isolates (70.4%) carrying all six tested virulence determinants (*stn*, *iroN*, *pefA*, *spvB*, *spvC*, and *spvR*). These findings align with previous studies that identified *S*. Enteritidis as the predominant NTS serovar responsible for human salmonellosis in Iran and other regions (Buddhasiri et al. 2023; Cao et al. 2023; Rezaei et al. 2022). The next most virulent serovar, *S*. typhimurium, exhibited a lower prevalence of virulence genes but harboured a higher frequency of ARGs. In contrast, non‐typed and *S*. Infantis isolates carried fewer plasmid‐encoded virulence determinants (*pefA*, *spvB*, *spvC*, and *spvR*) (Figures 2 and 3). Nonetheless, bacterial pathogenicity is also influenced by other factors, such as AMR and host susceptibility.

Finally, this study found that NTS isolates from the early stages of the poultry production chain showed lower antibiotic resistance than those from the later stages. However, NTS isolates recovered from slaughterhouses displayed higher levels of antibiotic resistance and harboured more antibiotic resistance genes, underscoring the need for improved hygiene and reduced human handling in slaughterhouses. Moreover, a subset of NTS isolates (non‐typed isolates) showed high levels of AMR. These isolates may represent diverse or emerging strains that were not detected by the multiplex PCR method used. Therefore, they should not be ignored in AMR surveillance. The majority of NTS isolates showed MDR profiles. Genetic analysis revealed that they contained diverse virulence and ARGs that could be transmitted via HGT. This evolutionary process can lead to more severe infections and contribute to the emergence of antibiotic resistance, posing a significant threat to human health.

## 5 Conclusion

This study offers a helpful perspective on the presence of heterogeneous NTS serovars with MDR profiles that contaminated poultry products, which is a considerable threat to human health in this region. Our results confirm the emergence of *S*. Infantis in the poultry industry and suggest that this serovar should be included in programs for monitoring and control of *Salmonella* throughout the poultry production chain. Although fluoroquinolone antibiotics are highly effective against NTS serovars, the use of antibiotics in both human and veterinary medicine must be strictly regulated. Producing safe poultry products requires the implementation of strict biosecurity measures, adherence to proper hygiene practices, and the use of automated slaughtering systems. Moreover, in 22% of the isolates (non‐typed *Salmonella*), serovar identification was not possible due to limitations of the diagnostic methods used. Therefore, we suggest continuous monitoring of *Salmonella* infections in the poultry industry using conventional methods in conjunction with highly discriminatory power methods (such as whole‐genome sequencing) to ensure a more accurate evaluation of NTS infections, serovar distribution, antibiotic resistance, and their implications for public health.

## Author Contributions

**Ali Zavari**: writing – review and editing, investigation, methodology, validation, analysis of the data, software, writing – original draft. **Mahdi Askari Badouei**: supervision, writing – review and editing, methodology, writing – original draft. **Gholamreza Hashemi Tabar**: project administration, writing – review and editing, methodology, funding acquisition, conceptualization.

## Funding

This Research was supported by Ferdowsi University of Mashhad, Mashhad, Iran, Grant No. 54042. (2021‐01‐26).

## Ethics Statement

The authors have nothing to report.

## Conflicts of Interest

The authors declare no conflicts interest.

## Supporting information




**Supplementary S1** provides detailed information on non‐typhoidal *Salmonella* isolates, including their sources, year of isolation, molecular serovar typing results, and profiles of multi‐drug resistance.

**Supplementary S2** includes the sequences of dual‐labelled probe and primers, targeted genes, annealing temperatures, and amplicon sizes used in the study.

## Data Availability

Data will be made available on request.
