# Supplementary material for: Molecular Epidemiology and Genetic Profile of Non‐Typhoidal Salmonella Serovars Isolated From Poultry Production Chain in Khorasan Province, Iran: A Comprehensive Analysis of Prevalence, Virulence Genes, and Antibiotic Resistance
Source: Vet Med Sci. 2026 Jan 6;12(1):e70757. doi: 10.1002/vms3.70757 (PMC12774793; doi:10.1002/vms3.70757)
Supplement: Supplementary file 2 — Supplementary S2 includes the sequences of dual‐labelled probe and primers, targeted genes, annealing temperatures, and amplicon sizes used in the study. [file VMS3-12-e70757-s001.docx]

**Title**: Molecular Epidemiology and Genetic Profile of Non-Typhoidal Salmonella Serovars Isolated from Poultry Sources in Khorasan Province, Iran: A Comprehensive Analysis of Prevalence, Virulence Genes, and Antibiotic Resistance

**Table. 1** List of primers sequence, PCR annealing temperature, amplicon size and target genes used in this study

| **primer** | **Primer sequnses** | **Annealing temprature** | **product size** | **Target genes** | **Refrences** |
| --- | --- | --- | --- | --- | --- |
| Primers and probe sequences used for Real time PCR | | | | | |
| Styinva-2-L | TCGTCATTCCATTACCTACC | 55 | 119 bp | *Salmonella enterica (invA)* | (1) |
| Styinva-2-R | AAACGTTGAAAAACTGAGGA |  |  |  |  |
| Styinva-2-P | 5′ FAM-TCTGGTTGATTTCCTGATCGCA -TAMRA3′ |  |  |  |  |
| Primers sequences used for Multiplex PCR | | | | | |
| *fliC* F | CCCCGCTTACAGGTGGACTAC | 58 | 433 bp | *Salmonella* Typhimurium  *(fliC)* | (2) |
| *fliC* R | AGCGGGTTTTCGGTGGTTGT |  |  |  |  |
| Prot6e F | ATATCGTCGTTGCTGCTTCC | 58 | 206 bp | *Salmonella* Enteritidis *(Prot6E)* | (3) |
| Prot6e R | CATTGTTCCACCGTCACTTTG |  |  |  |  |
| 558f | AACAACGACAGCTTATGCCG | 58 | 727 bp | *Salmonella* Infantis *(fljB)* | (4) |
| 1275r | CCACCTGCGCCAACGCT |  |  |  |  |
| Primers sequences used for Virulence genes identification | | | | | |
| spvB-F | CTATCAGCCCCGCACGGAGAGCAGTTTTTA | 60 | 717 bp | *spvB* | (5) |
| spvB-R | GGAGGAGGCGGTGGCGGTGGCATCATA |  |  |  |  |
| spvC-F | ACTCCTTGCACAACCAAATGCGGA | 60 | 571 bp | *spvC* | (6) |
| *spvC-R* | TGTCTCTGCATTTCGCCACCATCA |  |  |  |  |
| *spvR-F* | CAGGTTCCTTCAGTATCGCA | 57 | 306 bp | *spvR* | (7) |
| *spvR-R* | TTTGGCCGGAAATGGTCAGT |  |  |  |  |
| *stn* F | CTTTGGTCGTAAAATAAGGCG | 60 | 260 bp | enterotoxin | (8) |
| *stn* R | TGCCCAAAGCAGAGAGATTC |  |  |  |  |
| *iroN* F | ACTGGCACGGCTCGCTGTCGCTCTAT | 60 | 1205 bp | siderophore receptor | (5) |
| *iroN* R | CGCTTTACCGCCGTTCTGCCACTGC |  |  |  |  |
| *pefA*-F | ACACGCTGCCAATGAAGTGA | 60 | 456 bp | Major fimbrial subunit | (7) |
| *pefA*-R | ACTGCGAAAGATGCCACAGA |  |  |  |  |
| Primers sequences used for antimicrobial resistance genes identification | | | | | |
| *dhfrV*-F | CTGCAAAAGCGAAAAACGG | 60 | 432 bp | *dhfrV* | (9) |
| *dhfrV*-R | AGCAATAGTTAATGTTTGAGCTAAAG |  |  |  |  |
| *bla*_TEM_-F | CATTTCCGTGTCGCCCTTATTC | 57 | 800 bp | *bla*_TEM_ | (10) |
| *bla*_TEM_-R | CGTTCATCCATAGTTGCCTGAC |  |  |  |  |
| *bla*_SHV_-F | AGCCGCTTGAGCAAATTAAAC | 57 | 713bp | *bla*_SHV_ | (10) |
| *bla*_SHV_-R | ATCCCGCAGATAAATCACCAC |  |  |  |  |
| *Bla*_OXA_-F | GGCACCAGATTCAACTTTCAAG | 57 | 564bp | *bla*_OXA_ | (10) |
| *Bla*_OXA_-R | GACCCCAAGTTTCCTGTAAGTG |  |  |  |  |
| *tetA*-1 | GTAATTCTGAGCACTGTCGC | 60 | 950 bp | *tetA* | (11) |
| *tetA*-2 | CTGCCTGGACAACATTGCTT |  |  |  |  |
| *tetK*-1 | GTAGCGACAATAGGTAATAGT | 55 | 360 bp | *tetK* | (12) |
| *tetK*-2 | GTAGTGACAATAAACCTCCTA |  |  |  |  |
| *aadA*-F | TGATTTGCTGGTTACGGTGAC | 52 | 284 bp | *aadA* | (13) |
| *aadA*-R | CGCTATGTTCTCTTGCTTTTG |  |  |  |  |
| *sulI-F* | TTCGGCATTCTGAATCTCAC | 52 | 822 bp | *Sul1* | (9) |
| *sulI-R* | ATGATCTAACCCTCGGTCTC |  |  |  |  |

Refrences

1. Hoorfar J, Ahrens P, Rådstro¨m AP, Rådstro¨m R. Automated 5 Nuclease PCR Assay for Identification of Salmonella enterica. JOURNAL OF CLINICAL MICROBIOLOGY. 2000.

2. Arkali A, Çetinkaya B. Molecular identification and antibiotic resistance profiling of Salmonella species isolated from chickens in eastern Turkey. BMC Vet Res. 2020 Jun 19;16(1).

3. Malorny B, Bunge C, Helmuth R. A real-time PCR for the detection of Salmonella Enteritidis in poultry meat and consumption eggs. J Microbiol Methods. 2007 Aug;70(2):245–51.

4. Kardos G, Farkas T, Antal M, Nógrády N, Kiss I. Novel PCR assay for identification of Salmonella enterica serovar Infantis. Lett Appl Microbiol. 2007;45(4):421–5.

5. Skyberg JA, Logue CM, Nolan LK. Virulence genotyping of Salmonella spp. with multiplex PCR. Avian Dis. 2006 Mar;50(1):77–81.

6. Chiu CH, Su LH, Chu CH, Wang MH, Yeh CM, Weill FX, et al. Detection of multidrug-resistant Salmonella enterica serovar typhimurium phage types DT102, DT104, and U302 by multiplex PCR. J Clin Microbiol. 2006 Jul;44(7):2354–8.

7. Pasmans F, Van Immerseel F, Heyndrickx M, Martel A, Godard C, Wildemauwe C, et al. Host adaptation of pigeon isolates of Salmonella enterica subsp. enterica serovar Typhimurium variant Copenhagen phage type 99 is associated with enhanced macrophage cytotoxicity. Infect Immun. 2003 Oct 1;71(10):6068–74.

8. McWhorter AR, Davos D, Chousalkar KK. Pathogenicity of Salmonella strains isolated from egg shells and the layer farm environment in Australia. Appl Environ Microbiol. 2015;81(1):405–14.

9. Maynard C, Fairbrother JM, Bekal S, Sanschagrin F, Levesque RC, Brousseau R, et al. Antimicrobial resistance genes in enterotoxigenic Escherichia coli O149: K91 isolates obtained over a 23-year period from pigs. Antimicrob Agents Chemother. 2003 Oct 1;47(10):3214–21.

10. Dallenne C, da Costa A, Decré D, Favier C, Arlet G. Development of a set of multiplex PCR assays for the detection of genes encoding important β-lactamases in Enterobacteriaceae. Journal of Antimicrobial Chemotherapy. 2010 Jan 12;65(3):490–5.

11. Sáenz Y, Briñas L, Domínguez E, Ruiz J, Zarazaga M, Vila J, et al. Mechanisms of resistance in multiple-antibiotic-resistant Escherichia coli strains of human, animal, and food origins. Antimicrob Agents Chemother. 2004 Oct;48(10):3996–4001.

12. Strommenger B, Kettlitz C, Werner G, Witte W. Multiplex PCR assay for simultaneous detection of nine clinically relevant antibiotic resistance genes in Staphylococcus aureus. J Clin Microbiol. 2003 Sep 1;41(9):4089–94.

13. Yu ZN, Wang J, Ho H, Wang YT, Huang SN, Han RW. Prevalence and antimicrobial-resistance phenotypes and genotypes of Escherichia coli isolated from raw milk samples from mastitis cases in four regions of China. J Glob Antimicrob Resist. 2020 Sep 1;22:94–101.
